# Highly efficient cyclosarin degradation mediated by a β-cyclodextrin derivative containing an oxime-derived substituent

**DOI:** 10.3762/bjoc.7.182

**Published:** 2011-11-22

**Authors:** Michael Zengerle, Florian Brandhuber, Christian Schneider, Franz Worek, Georg Reiter, Stefan Kubik

**Affiliations:** 1 Fachbereich Chemie - Organische Chemie, Technische Universität Kaiserslautern, Erwin-Schrödinger-Straße, D-67663 Kaiserslautern, Germany, Fax: +49-631-205-3921; 2 Institut für Pharmakologie und Toxikologie der Bundeswehr, Neuherbergstraße 11, D-80937 München, Germany

**Keywords:** acetylcholinesterase, cyclodextrins, cyclosarin, neurotoxic organophosphonates, oximes

## Abstract

The potential of appropriately substituted cyclodextrins to act as scavengers for neurotoxic organophosphonates under physiological conditions was evaluated. To this end, a series of derivatives containing substituents with an aldoxime or a ketoxime moiety along the narrow opening of the β-cyclodextrin cavity was synthesized, and the ability of these compounds to reduce the inhibitory effect of the neurotoxic organophosphonate cyclosarin on its key target, acetylcholinesterase, was assessed in vitro. All compounds exhibited a larger effect than native β-cyclodextrin, and characteristic differences were noted. These differences in activity were correlated with the structural and electronic parameters of the substituents. In addition, the relatively strong effect of the cyclodextrin derivatives on cyclosarin degradation and, in particular, the observed enantioselectivity are good indications that noncovalent interactions between the cyclodextrin ring and the substrate, presumably involving the inclusion of the cyclohexyl moiety of cyclosarin into the cyclodextrin cavity, contribute to the mode of action. Among the nine compounds investigated, one exhibited remarkable activity, completely preventing acetylcholinesterase inhibition by the (−)-enantiomer of cyclosarin within seconds under the conditions of the assay. Thus, these investigations demonstrate that decoration of cyclodextrins with appropriate substituents represents a promising approach for the development of scavengers able to detoxify highly toxic nerve agents.

## Introduction

Cyclodextrins, cyclic oligosaccharides composed of α-1,4-linked D-glucose units, represent one of the most important classes of host systems in supramolecular chemistry [1]. Their easy availability, their ability to include organic nonpolar molecules into the cavity made up by the cyclically arranged glucose units in aqueous solution, their predictable and controllable binding properties, and their relatively straightforward chemical modification have made cyclodextrins indispensable tools in applications such as sensing [2], nanotechnology [3–4], polymer chemistry [5–8], medicinal chemistry [9–10], food chemistry [11], and others. Importantly, the scope of cyclodextrins goes beyond molecular recognition since the recognition event can in some cases be coupled with the chemical transformation of a substrate. This property was already realized in 1959 when it was shown that native cyclodextrins accelerate the cleavage of some acetic acid esters [12]. Subsequent work then established cyclodextrins containing appropriate substituents or dimeric cyclodextrins as a potent class of enzyme mimics [13–14]. Interestingly, it was also demonstrated relatively early in the field of cyclodextrin chemistry that native cyclodextrins are able to accelerate the cleavage of phosphates and phosphonates [15–18], including the highly neurotoxic organophosphonates (OP) sarin and soman [19–21]. While α-cyclodextrin, the cyclodextrin containing six anhydroglucose units along the ring, was shown to be most effective for sarin [17,22], the larger β-cyclodextrin with the seven-membered ring was demonstrated to also mediate soman degradation [19–20]. Moreover, the cyclodextrins were shown to act enantioselectively, being more effective for the more toxic (*R*)-(−)-enantiomer of sarin, for example [17,22]. Surprisingly, this work has largely been overlooked despite the fact that it provided strong indications for the potential applicability of cyclodextrins for the detoxification of chemical warfare agents. Only very recently has the use of cyclodextrins to induce degradation of neurotoxic OPs been addressed again. These investigations showed that β-cyclodextrin derivatives with a substituent along the wider rim of the cavity, bearing a nucleophilic group in the form of an iodosylbenzoate [23–25] or an oxime [26], efficiently react with paraoxon, cyclosarin (**GF**), and tabun thus reducing the inhibitory effects of these OPs on the key target of OP toxicity, acetylcholinesterase (AChE). Moreover, the results indicate that the mode of action of these cyclodextrin derivatives involves the formation of an inclusion complex with the OP.

**Figure 1:**
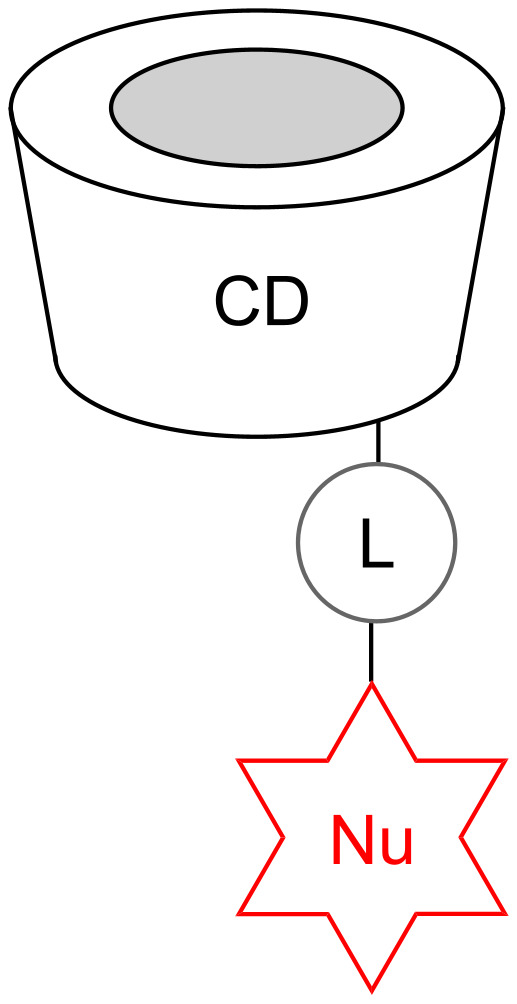
Schematic representation of the general structural design of the investigated cyclodextrin derivatives (CD = cyclodextrin ring, L = linking unit, Nu = reactive nucleophilic unit).

**Scheme 1:**
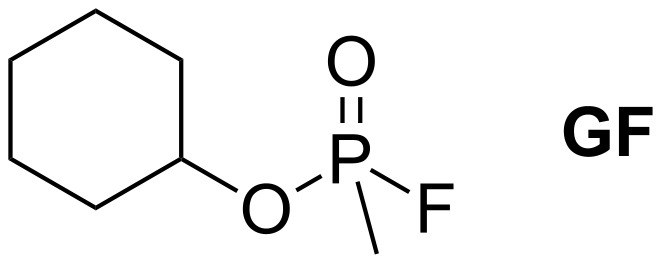
Structure of cyclosarin (**GF**).

The question thus arises as to whether suitable cyclodextrin derivatives could also be used in vivo as antidotes against OP poisonings. Such compounds should be able to act as scavengers by rapidly decomposing the OP into nontoxic byproducts before inhibition of AChE occurs. Catalytic action of the scavenger is desirable, reducing the necessary dose of the drug, but is not required if the toxicity is low. Since the amount of data currently available is too low to assess whether this approach has a realistic prospect of success, we initiated a research program aimed at the synthesis of a large number of structurally diverse cyclodextrin derivatives and the evaluation of their effect on OP degradation. In terms of structure, these compounds follow a common design principle, involving three distinct subunits each of which has a characteristic function (Figure 1):

- A cyclodextrin ring, which forms the basis of each compound. Complex formation between this subunit and the substrate should bring the P-atom of the substrate into spatial proximity with the substituent on the ring, thus facilitating the attack by the reactive group on the substituent. The type of cyclodextrin in this subunit (α, β, γ) controls the substrate affinity.
- The linking unit between the cyclodextrin ring and the reactive part of the substituent. This group should be chosen to allow straightforward synthetic access to the cyclodextrin derivatives, ideally allowing the synthesis to proceed in a modular fashion.
- The reactive unit bearing a functional group that should be able to specifically cleave the P–X bond on the substrate. In the case of **GF** (Scheme 1), for example, the most labile P–F bond is the one that is most prone to cleavage. In general, the functional group contains a suitable nucleophilic center, with α-effect nucleophiles possessing particularly promising activities [27–28]. Accordingly, the first step of OP deactivation is expected to consist of the phosphonylation of this nucleophilic group, similar to the phosphonylation of the serine residue in the active site of AChE during OP-mediated inhibition of this enzyme. If the compound thus formed is hydrolytically unstable, it will be cleaved in the aqueous medium, releasing the original reactive unit and allowing it to mediate another reaction.

Here, we describe our first results in this project, involving a series of β-cyclodextrin derivatives with substituents on the primary face of the cyclodextrin ring, containing oximes as nucleophilic groups. Oximes are well-known antidotes for the treatment of OP poisonings. Their mode of action involves reactivation of the OP-inhibited acetylcholinesterase [29], yet previous work has also indicated that certain oximes are able to cleave OPs directly [30]. We show that some of our cyclodextrin derivatives efficiently reduce **GF** concentrations in solution under physiological conditions within seconds, thus preventing the OP from inhibiting AChE. The observed correlation of structural and electronic parameters of the cyclodextrin derivatives with their activity strongly indicates that the interaction between the cyclodextrin ring and the substrate plays a decisive role in the mode of action.

## Results and Discussion

**Scheme 2:**
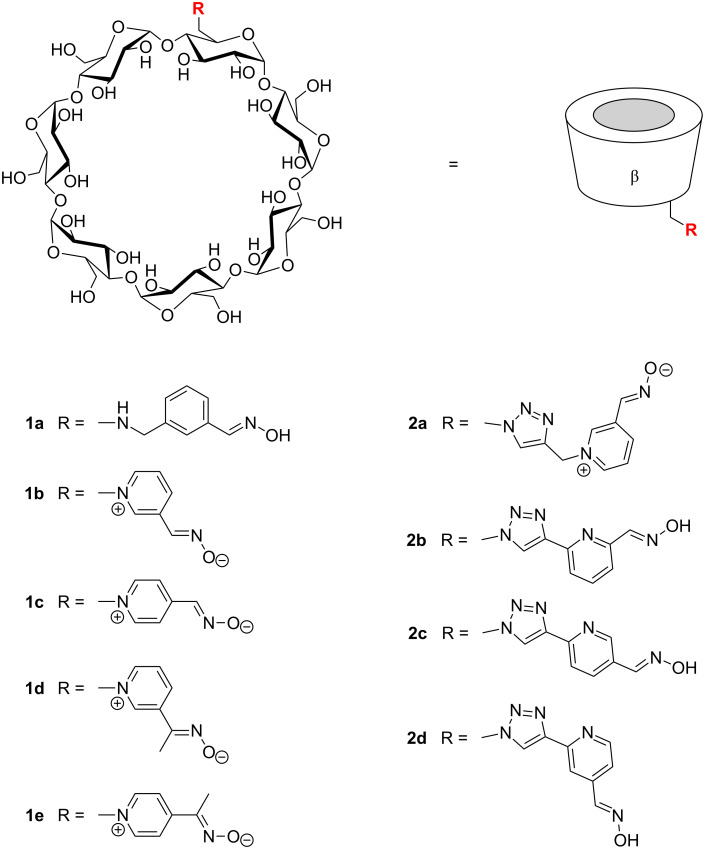
Cyclodextrin derivatives tested as potential **GF** scavengers.

**Synthesis.** The cyclodextrin derivatives investigated in this study are shown in Scheme 2. All compounds derive from the seven-membered β-cyclodextrin and contain the substituent in the 6-position of a glucose unit, i.e., along the narrower rim of the cyclodextrin cavity.

**Scheme 3:**
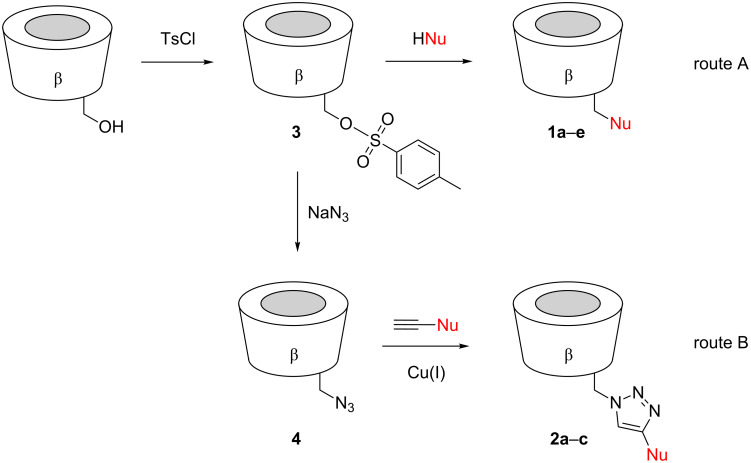
General strategies used for the preparation of the investigated cyclodextrin derivatives.

**Scheme 4:**
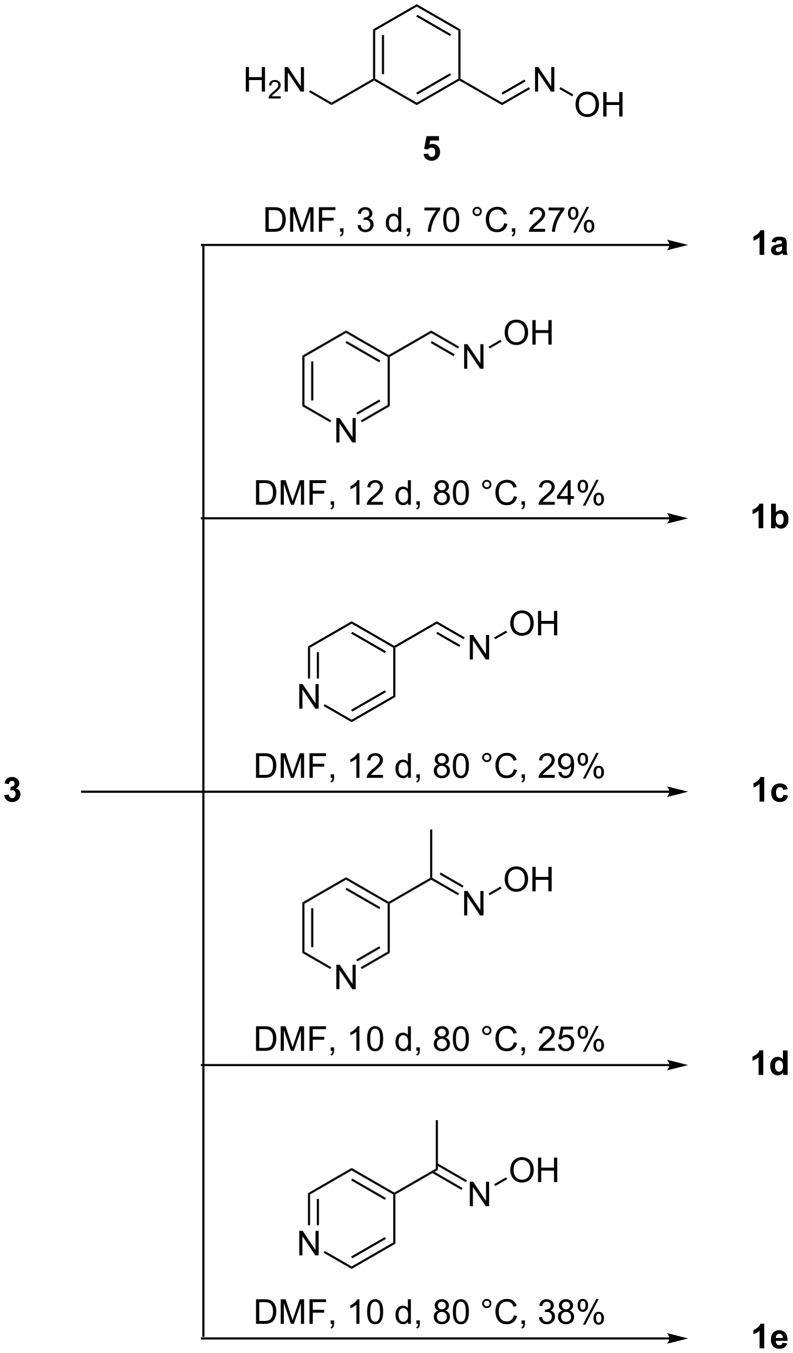
Reaction conditions used for the preparation of cyclodextrin derivatives **1a**–**e** from tosylate **3**.

**Scheme 5:**
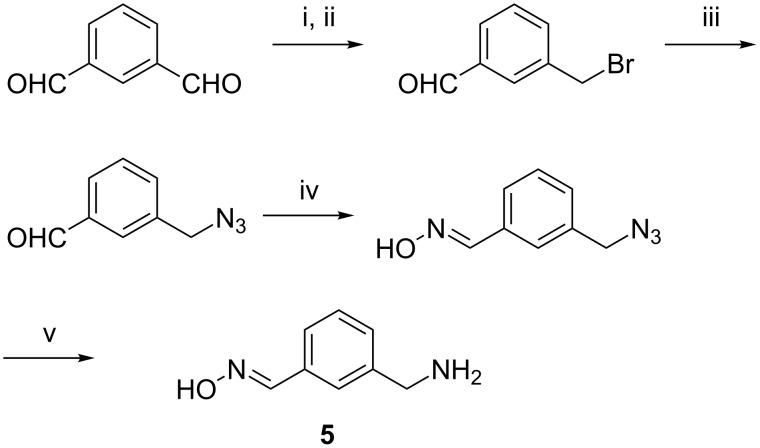
Synthesis of 3-(aminomethyl)benzaldehyde oxime (**5**). Reagents and conditions: i. NaBH_4_, EtOH, 1 h, 25 °C; ii. HBr/HOAc, 2 h, 25 °C, 38% (2 steps); iii. NaN_3_, EtOH, 4 h, reflux, 94%; iv. NH_2_OH·HCl, NEt_3_, EtOH, 1.5 h, 25 °C, 87%; v. PPh_3_, H_2_O/MeOH, 18 h, 25 °C, 80%.

These products were prepared by following two different routes. Cyclodextrins **1a**–**e** derive from mono-6-(*p*-tolylsulfonyl)-β-cyclodextrin (**3**), which can be expediently prepared from β-cyclodextrin and *p*-tolylsulfonyl chloride [31]. Reaction of **3** with an appropriate nucleophile then afforded the functionalized products (Scheme 3, route A). Compound **1a** was prepared by the reaction of **3** with 3-(aminomethyl)benzaldehyde oxime (**5**) (Scheme 4), the latter of which was synthesized from isophtalaldehyde, as shown in Scheme 5. Compounds **1b–e** were obtained from **3** and the corresponding pyridine aldoximes or ketoximes (Scheme 4) all of which are easily accessible from the commercially available aldehydes or ketones by reaction with hydroxylamine [32]. Attempts to also prepare the analogous derivative with the oxime group in the 2-position of the pyridinium ring, by reaction of **3** with 2-formylpyridine oxime or 2-acetylpyridine oxime, unfortunately failed to produce the desired products.

**Scheme 6:**
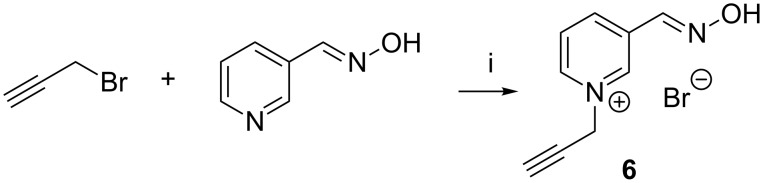
Synthesis of 3-((hydroxyimino)methyl)-1-(prop-2-ynyl)pyridinium bromide (**6**). Reagents and conditions: i. KI, DMF/toluene, 3 h, 25 °C, 39%.

**Scheme 7:**
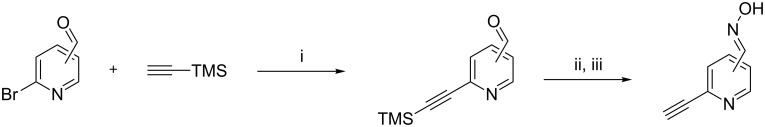
Syntheses of 6-ethynyl-formylpyridine oximes (**7a**–**c**). Reagents and conditions: i. CuI, (PPh_3_)_2_PdCl_2_, DPEphos, NEt_3_, 18–20 h, 25 °C, 71-87%; ii. NH_2_OH, H_2_O, 30 min, 80 °C; iii. TBAF, THF, 1 h, 0 °C, 68–83%.

**Scheme 8:**
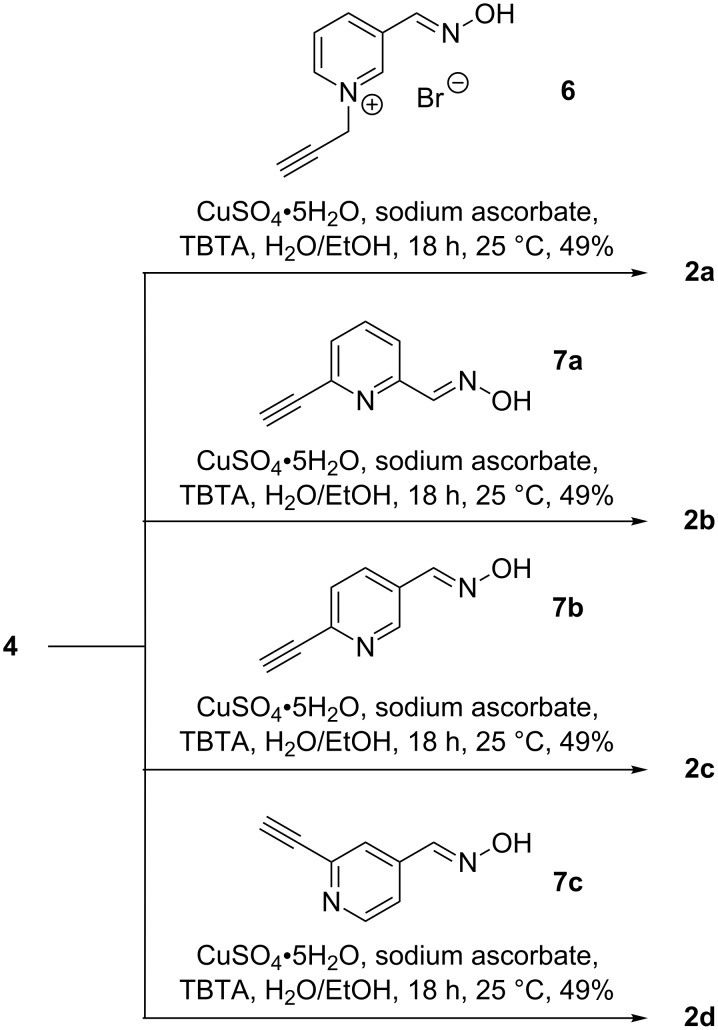
Reaction conditions used for the preparation of cyclodextrin derivatives **2a**–**d** from azide **4**.

The cyclodextrin derivatives **2a**–**d** contain 1,4-disubstituted 1,2,3-triazole moieties as the linking units. Accordingly, they were prepared by copper(I)-catalyzed azide–alkyne cycloaddition (“click-reaction”) [33] from mono-6-azido-6-deoxy-β-cyclodextrin (**4**) and a functionalized alkyne (Scheme 3, route B). Conjugations by copper(I)-catalyzed azide–alkyne cycloadditions have become popular in many different fields of chemistry [33–34], including cyclodextrin chemistry [35–41]. The alkyne **6** required for the synthesis of **2a** was obtained from propargyl bromide and 3-formylpyridine oxime (Scheme 6), and those for **2b**, **2c**, and **2d**, following the routes shown in Scheme 7. Reaction of these alkynes with **4** in the presence of copper(II) sulfate, sodium ascorbate and tris[(1-benzyl-1*H*-1,2,3-triazol-4-yl)methyl]amine (TBTA) [42] afforded the corresponding coupled products (Scheme 8).

All functionalized cyclodextrins **1a**–**e** and **2a**–**d** were purified by preparative HPLC. To this end, a universal method was developed that involves the use of a reversed-phase C18 column and a gradient of a binary solvent mixture, acetonitrile/0.025% aqueous ammonia. All products were thus obtained in analytically pure form, which is necessary to ensure that the subsequent evaluation of activity yields reliable results. Elemental analyses and NMR spectroscopy indicated that the pyridinium-containing cyclodextrins do not contain a counterion and that they therefore most probably represent the betaine forms depicted in Scheme 2.

**Figure 2:**
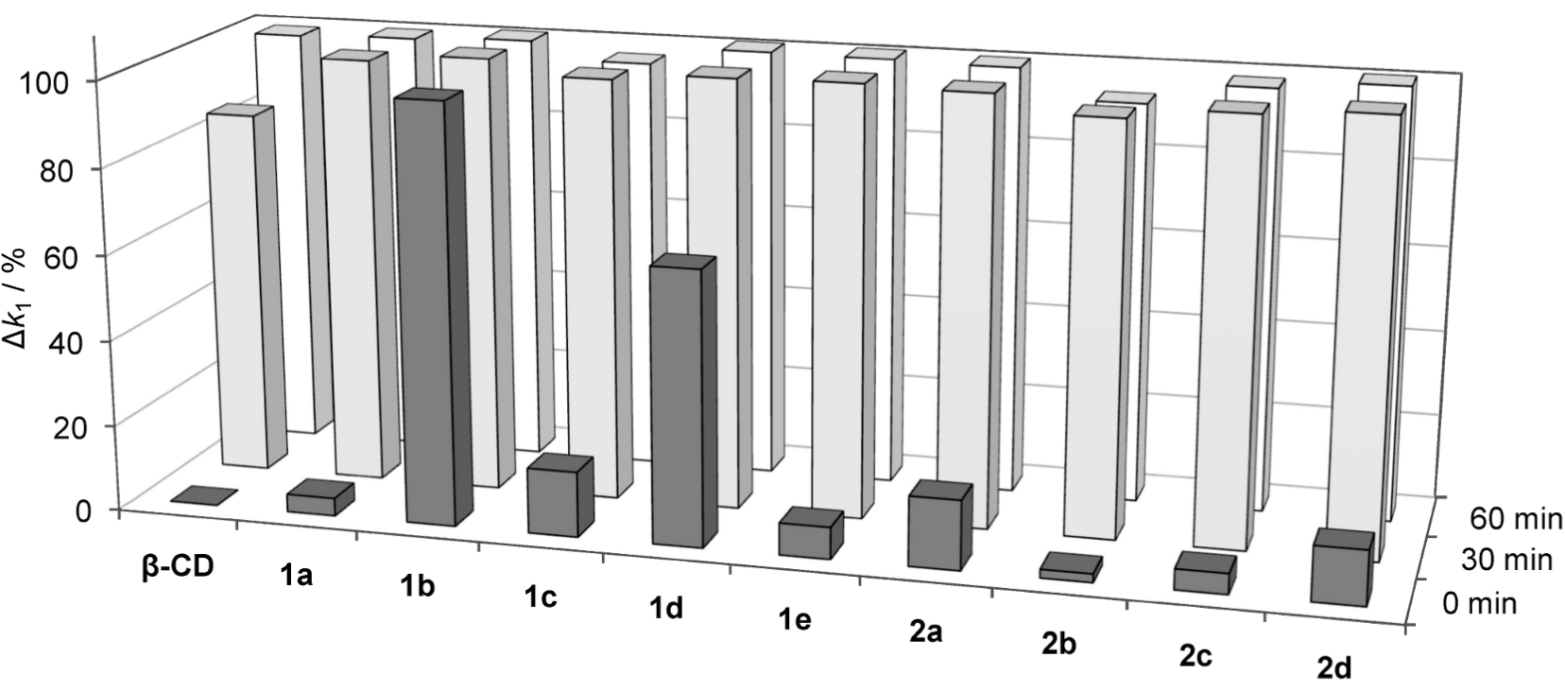
Diagram summarizing the observed Δ*k*_1_ values for cyclodextrins **1a**–**e** and **2a**–**d**. For comparison, the results obtained for native β-cyclodextrin are also included in the diagram. Large bars indicate low enzyme inhibition and, consequently, high activity of the corresponding cyclodextrin. The results are averages over three independent measurements.

**Qualitative assay.** In a first step, the ability of the prepared cyclodextrin derivatives to reduce **GF** concentration in solution and, as a consequence, the inhibitory effect of this OP on AChE was estimated by using a fully automated high-throughput screening assay recently developed for the characterization of potential nerve agent detoxifying materials [43]. This test involves incubation of the nerve agent with an excess of a respective cyclodextrin derivative at 37.0 °C in an aqueous TRIS-HCl buffer (pH 7.40). Aliquots of this solution were added to a solution containing human acetylcholinesterase (hAChE, EC 3.1.1.7), acetylthiocholine (ATCh) and 5,5'-dithiobis-(2-nitrobenzoic acid) (DTNB) immediately after mixing and after 30 and 60 min. The inhibitory effect of **GF** on the enzyme was then quantified photometrically by following the rate of formation of the 2-nitro-5-thiobenzoate dianion (Ellman assay) [44–45]. A first order rate constant *k*_1_ was derived from these curves by nonlinear regression analysis, which is a measure of the extent of enzyme inhibition: The larger the value of *k*_1_ the stronger the inhibitory effect of the nerve agent on the enzyme activity. By relating *k*_1_ to *k*_1_^ref^, the rate constant determined in the absence of the cyclodextrin, and to *k*_1_^native^, the rate constant determined in a preliminary assay in the absence of both cyclodextrin and **GF**, one obtains (*k*_1_^ref^−*k*_1_)/(*k*_1_^ref^−*k*_1_^native^)·100% = Δ*k*_1_, a term which correlates with the activity of the cyclodextrin. If the extent of enzyme inhibition is the same in the absence and the presence of the cyclodextrin (*k*_1_^ref^ = *k*_1_) the cyclodextrin is inactive and Δ*k*_1_ = 0%. If, however, the nerve agent is decomposed faster in the presence of the cyclodextrin than during the background reaction, *k*_1_ becomes smaller with respect to *k*_1_^ref^ until it approaches the value of *k*_1_^native^. As a consequence Δ*k*_1_ increases up to 100% for full enzymatic activity (*k*_1_ = *k*_1_^native^). The results of this assay obtained for cyclodextrin derivatives **1a**–**e** and **2a**–**d** are summarized in Figure 2.

Figure 2 shows that even native β-cyclodextrin exhibits a substantial activity, consistent with the results of previous investigations [19–20]. The reduction of AChE inhibition is clearly visible after 30 min, and the enzyme is fully active when **GF** was incubated with β-cyclodextrin for 1 h prior to the Ellman assay. Notably, all substituted cyclodextrin derivatives already exhibit an effect in the first measurement, thus clearly demonstrating the enhancement of activity caused by the substituents. While this effect is small for most cyclodextrins, it is significant for **1d**, and the activity of **1b** is so high that no enzyme inhibition is observable even in the first measurement.

The correlation between the type of substituent on the cyclodextrin and the reduction of AChE inhibition, which is evidenced in Figure 2, indicates that the activity of these cyclodextrin derivatives depends sensitively on the structural parameters of the substituents in combination with the electronic properties. The generally larger activity of the pyridinium derivatives such as **1b**–**e** and **2a** with respect to compounds containing a neutral pyridine ring can most probably be attributed to the higher acidity of the aldoxime proton in pyridinium aldoximes, for example. For reference, the p*K*_a_ of the aldoxime proton in 3-formylpyridine oxime amounts to 10.36 and that of the corresponding proton in the 1-methiodide of 3-formylpyridine oxime to 9.22. Correspondingly, the p*K*_a_ of the aldoxime proton in 4-formylpyridine oxime (9.99) also decreases by more than one order of magnitude to 8.57 upon methylation of the ring nitrogen [46]. Thus, oximes on pyridinium rings are deprotonated more easily, which renders them more nucleophilic.

The influence of structural effects on the activity becomes evident when comparing **1b** and **2a**, both of which have the oxime moiety in the same position on the aromatic ring. The significantly larger activity of **1b** is an indication that the positioning of the nucleophilic group closer to the cavity of the cyclodextrin facilitates the reaction with **GF**. Interestingly, even small structural variations such as shifting the substituent on the pyridinium ring from the 3- into the 4-position (**1b** versus **1c**) or replacing the aldoxime with a ketoxime (**1b** versus **1d**) are associated with a significant loss of activity. While the latter effect is presumably due to the lower acidity of ketoximes with respect to aldoximes by ca. one order of magnitude [32], the higher activity of **1b** with respect to **1c** is more likely to be a structural effect, since on the basis of the p*K*_a_ values of 1-methiodides of 3-formylpyridine oxime (9.22) and 4-formylpyridine oxime (8.57) one would expect the opposite trend.

The pronounced activity of the investigated cyclodextrin derivatives, in particular of **1b**, also indicates that noncovalent interactions, most probably the inclusion of the apolar cyclohexyl moiety of **GF** into the cyclodextrin cavity, are an important aspect of the mode of action. Assuming that the affinity of **GF** to β-cyclodextrin in water is similar to that of cyclohexanol (687 M^−1^ at 30 °C) [47–48], and that it is approximately independent of the type of substituent, ca. 25% of the substrate molecules are estimated to reside in the cyclodextrin cavity under the conditions of the assay (500 μM cyclodextrin and 1 μM **GF**). This value presumably represents a lower limit, because hydrogen-bonding interactions between the OH-groups along the rim of the cyclodextrin cavity and the P=O group of the substrate can cause the **GF**complex to be more stable than that of cyclohexanol. However, it indicates that complexation of only part of the total substrate amount could ensure efficient conversion, if the oxime group of the cyclodextrin can approach the phosphonate moiety of the substrate in the complex, and if complexation/decomplexation kinetics are fast as is usually the case for cyclodextrin complexes [49]. The remarkable high activity observed for **1b** prompted us to evaluate in more detail the rate with which **GF** is degraded in the presence of this compound. For comparison, the less active isomer of **1b**, 4-substituted derivative **1c**, and the triazole-linked derivative **2a** were also included in this study.

**Figure 3:**
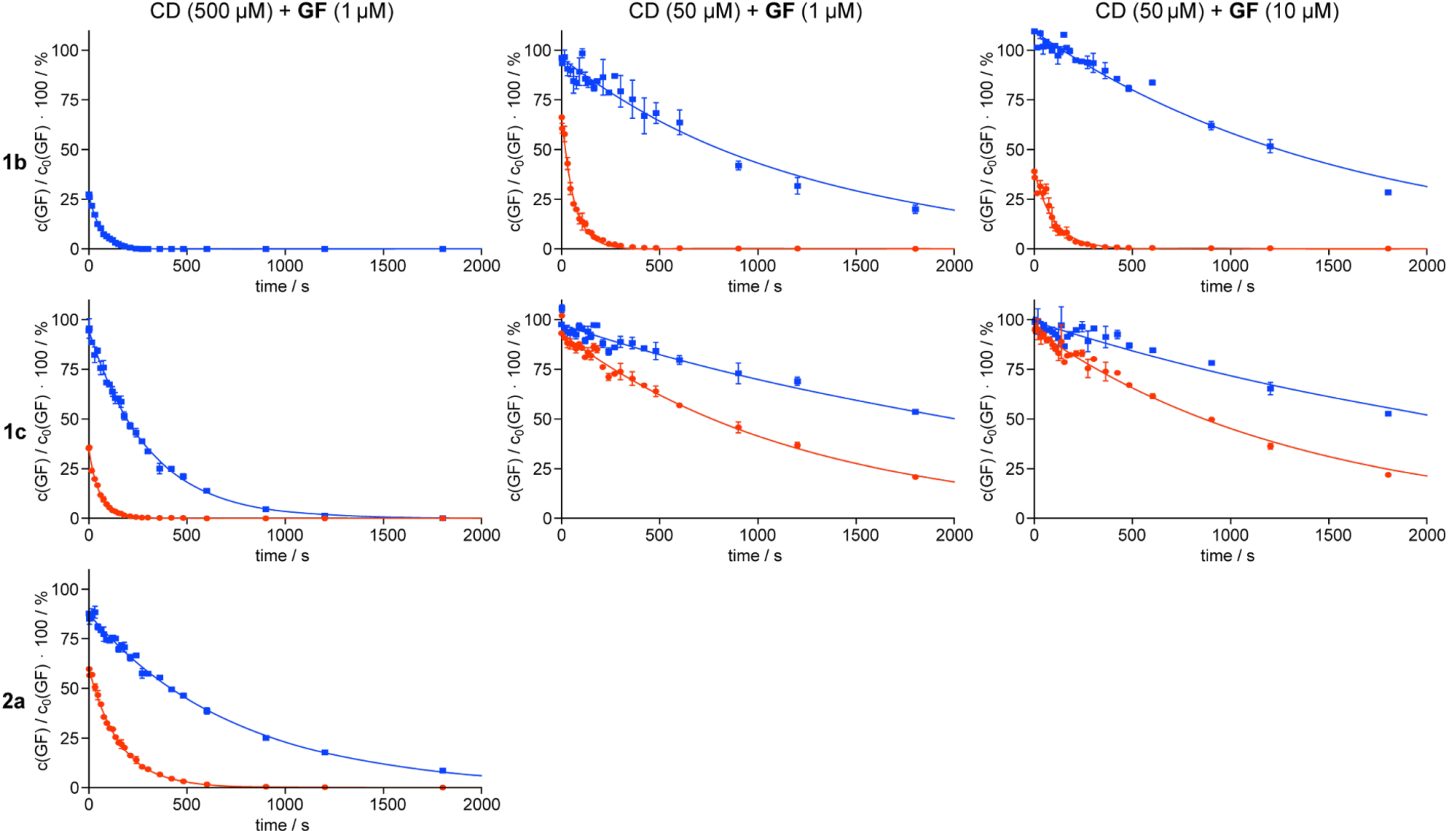
Time-dependent decrease of **GF** concentration in the presence of **1b** (top row), **1c** (middle row), and **2a** (bottom row). The measurements were performed at different concentrations of cyclodextrin (CD) and nerve agent, which are indicated at the top of each column. The data points (means ± SD, *n* = 2) denote the experimental results normalized to the initial concentration of **GF**, c_0_(**GF**). The curves show the results of fitting the data points to a first-order rate reaction. Disappearance of the (−)-enantiomer of **GF** is shown in red and that of the (+)-enantiomer in blue.

**Quantitative assay.** The kinetic parameters for the reduction of **GF** concentration in solution mediated by **1b**, **1c**, and **2a** were determined as described previously for other cyclodextrin derivatives [23]. Briefly, a buffered solution (pH 7.40) containing the nerve agent was incubated at 37.0 °C and an aliquot was taken to determine the initial concentration of **GF**, c_0_(**GF**). After the addition of a thermostatted cyclodextrin solution, aliquots were taken at defined intervals. These samples were immediately extracted with hexane and subjected to GC–MS analysis by using *d*_11_-**GF** (**GF** with a perdeuterated cyclohexyl residue) as the internal standard. The use of a chiral stationary phase allowed independent evaluation of the effect of the cyclodextrin on both **GF** enantiomers. The results of these measurements are shown in Figure 3.

One important aspect that becomes immediately apparent when looking at the graphs in Figure 3 is that the cyclodextrins act enantioselectively, with the (−)-enantiomer of **GF** being the one that disappears faster, independent of the cyclodextrin derivative used. This result, which is consistent with those of previous investigations [22–23], is another indication of the involvement of the chiral cyclodextrin residue in the reaction. The top left graph shows that under the standard conditions of this measurement (−)-**GF** is consumed so quickly, with conversion being complete after ca. 5 s, that no reliable kinetic analysis could be performed. Decomposition of the corresponding (+)-enantiomer is slower, but also complete after ca. 4 min. In order to follow the rate with which (−)-**GF** disappears, the concentration of cyclodextrin during incubation with **GF** was reduced to 50 μM. As expected, this modification of conditions caused a reduced rate of conversion for both enantiomers, presumably because the amount of **GF** molecules bound inside the cyclodextrin cavity decreases by a factor of ca. 10. Performing the analysis at 50 μM of cyclodextrin and 10 μM of **GF** does not have a large effect on the rate of reaction, which is consistent with the fact that increasing the **GF** concentration does not shift the complex equilibrium to a large extent. It should be noted that this assay currently does not allow us to ascertain whether the action of the cyclodextrins is stoichiometric or catalytic, because the cyclodextrin derivatives were used in excess with respect to **GF**.

Similar trends were observed for the other cyclodextrin derivatives, although their overall rates of conversion were consistently lower than those of **1b**. Quantitative information in terms of the observed rate constants *k*_obs_ and half-lives *t*_1/2_was obtained by fitting the experimentally obtained decay curves to a first order rate reaction and subtracting the effect of spontaneous **GF** hydrolysis under these conditions (1.5·10^−4^ s^−1^) [23]. The results thus obtained are summarized in Table 1.

**Table 1:** Kinetic constants determined for **GF** degradation mediated by cyclodextrins **1b**, **1c**, and **2a** (*k*_obs_ in s^−1^, *t*_1/2_ in s).

cyclodextrin concentration	cyclodextrin		(−)-**GF**	(+)-**GF**	(−)-**GF**	(+)-**GF**
			1 μM		10 μM	

500 μM	**1b**	*k*_obs_	n.d.^a^	1.6·10^−2^		
		*t*_1/2_	n.d.^a^	43		
	**1c**	*k*_obs_	1.8·10^−2^	3.1·10^−3^		
		*t*_1/2_	39	211		
	**2a**	*k*_obs_	6.1·10^−3^	1.2·10^−3^		
		*t*_1/2_	114	583		

50 μM	**1b**	*k*_obs_	1.5·10^−2^	6.5·10^−4^	9.6·10^−3^	4.7·10^−4^
		*t*_1/2_	46	1071	72	1465
	**1c**	*k*_obs_	6.6·10^−4^	1.8·10^−4^	5.9·10^−4^	1.7·10^−4^
		*t*_1/2_	1053	3857	1179	4143

^a^no reliable rate constant and half-life could be determined.

Comparison of these rate constants with ones obtained previously under similar conditions for native β-cyclodextrin and 2-*O*-(carboxy-iodosobenzyl)-β-cyclodextrin leads to the conclusion that the substituted cyclodextrins investigated here enhance the **GF** decomposition by at least one order of magnitude more efficiently than native β-cyclodextrin does [23]. Degradation of (+)-**GF** is ca. twice as fast in the presence of **1b** than in the presence of 2-*O*-(carboxy-iodosobenzyl)-β-cyclodextrin. Because of the relatively low enantioselectivity observed for 2-*O*-(carboxy-iodosobenzyl)-β-cyclodextrin, the activity of cyclodextrin **1b** is estimated to be at least one order of magnitude higher. More detailed information about the kinetics and thermodynamics of the reaction could be obtained by following the dependency of the rate of reaction on the **GF** concentration (Michaelis–Menten kinetics). These measurements are, however, demanding in light of the complexity of the currently used kinetic assay and have therefore not yet been performed.

## Conclusion

These investigations demonstrate that appropriately substituted cyclodextrin derivatives can efficiently reduce the inhibitory effect of **GF** on AChE under physiological conditions. The relatively strong effect of the cyclodextrin derivatives on **GF** degradation and, in particular, the observed enantioselectivity are good indications for noncovalent interactions between the cyclodextrin ring and the substrate, presumably involving the inclusion of the cyclohexyl moiety of cyclosarin into the cyclodextrin cavity, which contribute to the mode of action. In addition, the correlation between structural parameters and activity can be rationalized on the basis of the distance of the substituents from the cyclodextrin cavity, where OP binding presumably takes place, and the expected nucleophilicity of the oxime groups of the substituents. With **1b** a compound could be identified that, to the best of our knowledge, currently represents the most active cyclodextrin derivative mediating **GF** degradation in solution. These results make us optimistic that substituted cyclodextrins represent a very promising platform for the development of scavengers for highly toxic organophosphonates, including also ones that are more persistent than **GF**, such as tabun or VX. Work in this context is ongoing in our group.

## Experimental

**General details**. The synthesized compounds were characterized as follows: Melting points, Müller SPM-X 300; NMR, Bruker Avance 600, Bruker DPX 400; MALDI-TOF-MS, Bruker Ultraflex TOF/TOF; ESI–MS, Bruker Esquire 3000; IR, FT-IR System Spectrum BX, Perkin-Elmer; elemental analysis, Elementar vario Micro cube. All chemicals, unless other stated, are commercially available and were used without further purification. Cyclosarin and deuterated cyclosarin (*d*_11_-cyclosarin, *d*_11_-**GF**) (>98% by GC–MS, ^1^H NMR, and ^31^P NMR) were made available by the German Ministry of Defense (All experiments with cyclosarin were performed at the Institut für Pharmakologie und Toxikologie der Bundeswehr). Hemoglobin-free erythrocyte ghosts as a source for human erythrocyte acetylcholinesterase (AChE, EC 3.1.1.7) were prepared according to the procedure of Dodge et al. [50] with minor modifications [51]. AChE activity was adjusted to 4000 U/l by dilution with phosphate buffer (0.1 M, pH 7.40). Aliquots were stored at a temperature of −80 °C. Prior to use, ghosts were homogenized with a Sonoplus HD 2070 ultrasonic homogenator (Bandelin electronic, Berlin, Germany) twice for 5 s with a 20 s interval to achieve a homogeneous matrix. For the preparative HPLC the following conditions were used: HPLC, Dionex UltiMate 3000; column, Thermo Fisher, BetaBasic-18, 250 × 21.2 mm, 5 μm particle size; flow, 12 mL/min; eluent, aqueous: 0.025% aqueous ammonia, organic: acetonitrile; for the separation of neutral compounds (**1a**, **2b**–**2d**) the following gradient was used: 0–6 min, 0% organic; 6–27 min, linear increase of organic to 40%; 27–33 min, 40% organic; 33–39 min, linear decrease to 0% organic; 39–45 min, 0% organic; charged compounds (**1b**–**1e**, **2a**) were purified by employing a slightly different gradient: 0–6 min, 0% organic; 6–22 min, linear increase to 15% organic; 22–25 min, linear increase to 50% organic; 25–30 min, 50% organic; 30–37 min, linear decrease to 0% organic; 37–45 min, 0% organic.

**Scheme 9:**
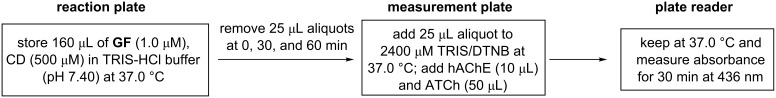
Schematic protocol for the qualitative assay.

**Qualitative assay.** The qualitative test was performed by using a Tecan Freedom EVO liquid handling system (Männedorf, Switzerland) [43]. A solution of cyclodextrin (500 µM) and **GF** (1 µM) was prepared and incubated in TRIS-HCl buffer (0.1 M, pH 7.40) at 37.0 °C (Scheme 9). Immediately after mixing, a sample (25 µL) was transferred to a measuring plate prefilled with buffer (2400 µL, 0.1 M TRIS-HCl, pH 7.40 and 0.3 mM DTNB) and human AChE (10 µL) and preheated to 37.0 °C. ATCh (50 µL, 49.7 mM) was added immediately, the microplate was transferred to a photometer and the absorption was measured at 436 nm for 30 min while the temperature was maintained at 37.0 °C. Further aliquots of the test mixture were taken after 30 min and 60 min and treated analogously. The rate constants *k*_1_ were determined by nonlinear regression analysis of the resulting curves by using Prism 5.0 (GraphPad Software, San Diego, CA, USA) as described previously [43]. All results shown are mean values of *n* = 3 experiments. The value for *k*_1_^native^ was determined in independent experiments to be 8.56·10^−5^ s^−1^.

**Scheme 10:**
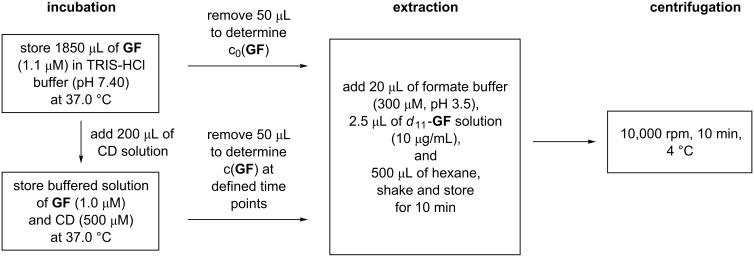
Schematic protocol of the quantitative assay.

**Quantitative assay.** The quantitative assay was performed in a stirred 2.0 ml cryo vial (Wheaton Science Products, Millville, NJ, USA) positioned in a temperature controlled water bath. Buffer (1850 µL, TRIS-HCl 0.1 M, pH 7.40) containing **GF** (1.1 µM) was incubated at 37.0 °C and an aliquot (50 µL) was removed to determine the initial concentration of **GF**, c_0_(**GF**) (Scheme 10). After preheated cyclodextrin solution (200 µL, final concentration 500 µM) was added, aliquots (50 µL) were taken at defined time points. These samples were transferred into a tube containing sodium formate buffer (20 µL, 300 µM, pH 3.5) and a solution of the internal standard *d*_11_-**GF** in 2-propanol (2.5 µL, 10 µg/mL). Afterward, ice-cold hexane (500 µL) was immediately added, and the mixture was shaken vigorously and stored on ice. Each sample was centrifuged as soon as possible (10.000 rpm, 10 min, 4 °C), and the organic layer was removed immediately and transferred to GC vials for analysis. The experimental results were normalized to the initial concentration of **GF** in the absence of cyclodextrin, c_0_(**GF**). The kinetic constants *k*_obs_ were determined by, first, nonlinear regression analysis of the resulting decay curves (using GraphPad Prism 5.0, San Diego, CA, USA) on the basis of the following equation: c_(+/-)_**_GF_**_,_*_t_* = c_(+/-)_**_GF_**_,0*_·exp(−*k*_obs_·*t*), in which c_(+/-)_**_GF_**_,_*_t_* denotes the concentration of (+)-**GF** or (−)-**GF** at a certain time *t* and c_(+/-)_**_GF_**_,0*_ the concentration of (+)-**GF** or (−)-**GF**immediately after cyclodextrin addition, followed by subtracting the effect of spontaneous **GF** hydrolysis (1.5·10^−4^ s^−1^) [23]. All data shown are the mean values of *n* = 2 experiments.

**Quantification of GF enantiomers by PCI MS.** Quantification of **GF** enantiomers was performed by GC–MS, as described before but with slight modifications [52]. In brief, a gas chromatographic system 6890N coupled with a mass spectrometer detector 5973 with positive chemical ionization (PCI) (Agilent Technologies, Waldbronn, Germany), large volume injection (LVI), and a GAMMA DEX^TM^ 225 GC column (30 m × 0.25 mm, 0.25 µm film thickness, Sigma–Aldrich Chemie, Taufkirchen, Germany) was used (Table 2). CI quantification was performed by applying ammonia 6.0 as reactant gas with a flow rate of 2.0 mL/min and helium as carrier gas with a flow rate of 1.3 mL/min. The following oven temperature program was applied: 50 °C for 4.5 min, increase to 100 °C at 40 °C/min and subsequently to 135 °C at 3 °C/min, maintain this temperature for 2 min, further increase to 170 °C at 40 °C/min. The limit of quantification was estimated to be 2.5 pg per enantiomer.

**Table 2:** GC–MS parameters for PCI analysis of **GF**.

injection program		MS parameters	

injection volume	50 µL	detected mass	*m*/*z* 198, *m*/*z* 209
injection speed	20 µL/min	dwell time	125 ms
initial temperature	40 °C, hold for 0.05 min	solvent delay	13.00 min
initial end temperature	260 °C, hold for 2 min		
initial time	2.70 min		
vent time	2.60 min		
vent flow	10.0 mL/min		
purge time	4.60 min		

## Supporting Information

File 1 Detailed experimental procedures and physical data for all newly prepared compounds.
