# Supplementary material for: Highly efficient cyclosarin degradation mediated by a β-cyclodextrin derivative containing an oxime-derived substituent
Source: Beilstein J Org Chem. 2011 Nov 22;7:1543–54. doi: 10.3762/bjoc.7.182 (PMC3252857; doi:10.3762/bjoc.7.182)
Supplement: File 1 — Detailed experimental procedures and physical data for all newly prepared compounds. [file Beilstein_J_Org_Chem-07-1543-s001.pdf]

Supporting Information

for

Highly efficient cyclosarin degradation mediated by a β-cyclodextrin derivative containing an oxime-derived substituent

Michael Zengerle[1], Florian Brandhuber[2], Christian Schneider[1], Franz Worek[2], Georg Reiter[2], Stefan Kubik[*1]

Address: [1]Fachbereich Chemie - Organische Chemie, Technische Universität Kaiserslautern, Erwin-Schrödinger-Straße, D-67663 Kaiserslautern, Germany, Fax: +49-631-205-3921 and [2]Institut für Pharmakologie und Toxikologie der Bundeswehr, Neuherbergstraße 11, D-80937 München, Germany

Email: Stefan Kubik - kubik@chemie.uni-kl.de

*Corresponding author

Detailed experimental procedures and physical data for all newly prepared compounds

Content

3-(Azidomethyl)benzaldehyde S2
3-(Azidomethyl)benzaldehyde oxime S2
3-(Aminomethyl)benzaldehyde oxime (**5**) S2
6-(1-(3-(Hydroxyiminomethyl)phenyl)methylaminyl)-6-deoxy-β-cyclodextrin (**1a**) S2
Cyclodextrins **1b**–**1e** S3
3-((Hydroxyimino)methyl)-1-(prop-2-ynyl)pyridinium bromide (**6**) S3
General procedure for the preparation of 6-((trimethylsilyl)ethynyl)-formylpyridines S4
General procedure for the preparation of 6-ethynyl-formylpyridine oximes S5
Cyclodextrins **2a**–**2d** S6
References S8

3-(Azidomethyl)benzaldehyde. 3-Bromobenzaldehyde [1] (2.10 g, 10.6 mmol) was dissolved in ethanol (150 ml). Sodium azide (2.74 g, 42.2 mmol) was added and the resulting mixture was heated to reflux for 4 h. After the solvent was removed, the residue was dissolved in water, the aqueous phase was extracted with dichloromethane, the combined organic layers were dried over sodium sulfate, and the solvent was removed in vacuo. Yield: 1.61 g (94%); ^{1}H NMR ($CDCl_3$, 400 MHz) δ 4.45 (s, 2H), 7.56–7.58 (m, 2H), 7.84–7.86 (m, 2H), 10.03 (s, 1H); ^{13}C NMR ($CDCl_3$, 100.6 MHz) δ 54.2, 129.0, 129.7, 129.8, 134.0, 136.8, 137.0 192.0; IR (cm^{-1}) 1701 (ν (C=O)), 2102 (azide); elemental analysis calcd (%) for $C_8H_7N_3O$: C, 59.62; H, 4.38; N, 26.07; found: C, 59.44; H, 4.37; N, 25.94.

3-(Azidomethyl)benzaldehyde oxime. Hydroxylamin hydrochloride (1.39 g, 20.0 mmol) and triethylamine (3.04 ml, 22.0 mmol) were dissolved in ethanol (50 ml). After the addition of 3-azidobenzaldehyde (1.61 g, 10.0 mmol) the resulting reaction mixture was heated to reflux for 1.5 h. The solvent was removed and the residue was purified by column chromatography by using hexane/MTBE, 9:1 (v/v) as eluent. Yield: 1.26 g (72%); R_f 0.23 (hexane/MTBE, 9:1 (v/v)); ^{1}H NMR ($CDCl_3$, 400 MHz) δ 4.40 (s, 2H), 7.24–7.26 (m, 1H), 7.34–7.38 (m, 1H), 7.55 (s, 2H), 8.17 (s, 1H), 8.33 (s, 1H); ^{13}C NMR ($CDCl_3$, 100.6 MHz) δ 54.6, 126.7, 127.2, 129.5, 129.8, 132.8, 136.3, 150.1; IR (cm^{-1}) 2102 (azide); ESI-TOF MS, *m*/*z* (%) 176.9 (100%) $[M + H]^+$; elemental analysis calcd (%) for $C_8H_8N_4O$: C, 54.54; H, 4.58; N, 31.80; found: C, 54.33; H, 4.83; N, 31.73.

3-(Aminomethyl)benzaldehyde oxime (**5**). 3-(Azidomethyl)benzaldehyde oxime (1.26 g, 7.17 mmol) and triphenylphosphine (3.76 g, 14.3 mmol) were dissolved in methanol (50 ml). Water (258 mg, 14.3 mmol) was added and the resulting reaction mixture was stirred for 18 h at 25 °C. After the solvent was removed, the residue was washed with MTBE and recrystallized from hexane/ethyl acetate. Yield: 851 mg (79%); ^{1}H NMR ($CDCl_3$, 400 MHz) δ 1.82 (s, br, 2H), 3.72 (s, 2H), 7.29–7.33 (m, 2H), 7.39–7.42 (m, 1H), 7.55 (s, 1H), 8.10 (s, 1H), 11.22 (s, 1H); ^{13}C NMR (D_2O, 100.6 MHz) δ 45.5, 124.4, 125.0, 128.1, 128.5, 132.9, 144.8, 148.3; IR (cm^{-1}) 3286 (ν (NH)), 3364 (ν (NH)); ESI-TOF MS, *m*/*z* (%) 134.0 (26%) $[M – OH + H]^+$, 151.0 (100%) $[M + H]^+$; elemental analysis calcd (%) for $C_8H_{10}N_2O$: C, 63.98; H, 6.71; N, 18.65; found: C, 63.89; H, 6.74; N, 18.59.

6-(1-(3-(Hydroxyiminomethyl)phenyl)methylaminyl)-6-deoxy-β-cyclodextrin (**1a**). Mono-6-(*p*-tolylsulfonyl)-β-cyclodextrin (**3**) [2] (0.50 g, 0.4 mmol) and 3-(aminomethyl)benzaldehyde oxime (0.29 g, 1.94 mmol) were dissolved in dry DMF (5 ml). After being stirred at 70 °C under a nitrogen atmosphere for 3 d the reaction mixture was poured into acetone. The precipitate was filtered off and washed with acetone. Separation and

purification of the product was achieved by preparative HPLC. Yield: 133 mg (27%); ^{1}H NMR (DMSO-d_6, 400 MHz) δ 1.83 (m, 1H), 2.75 (m, 1H), 2.90 (m, 1H), 3.31–3.41 (m, 14H, beneath H_2O signal), 3.49–3.73 (m, 28H), 4.43–4.55 (m, 6H), 4.83 (s, 7H), 5.69–5.82 (m, 14H), 7.29–7.32 (m, 2H), 7.43–7.45 (m, 1H), 7.52 (s, 1H), 8.11 (s, 1H), 11.18 (s, 1H); ^{13}C NMR (DMSO-d_6, 100.6 MHz) δ 49.0, 52.9, 59.9, 70.7–73.1, 81.5–83.7, 102.0–102.3, 124.6, 125.8, 128.5, 128.7, 132.9, 141.6, 148.3; MALDI-TOF MS, *m*/*z* (%) 1251.7 (41%) $[M - O + H]^+$, 1267.8 (100%) $[M + H]^+$), 1289.8 (63%) $[M + Na]^+$, 1305.8 (17%) $[M + K]^+$; elemental analysis calcd (%) for $C_{50}H_{78}N_2O_{35}\cdot 2H_2O$: C, 46.08; H, 6.34; N, 2.15; found: C, 45.86; H, 6.26; N, 2.06.

Cyclodextrins 1b–1e. The pyridine aldoximes and ketoximes were prepared as described in the literature [3]. Specifically, hydroxylamine hydrochloride (10 mmol) and sodium hydroxide (10 mmol) were dissolved in water (20 ml) at 80 °C. After the addition of a formyl or an acetyl pyridinium derivative (5 mmol), stirring was continued for 1 h at 80 °C. The reaction mixture was cooled to 25 °C whereupon the respective product crystallized as colorless needles in a yield of 60–89%. The oximes thus prepared were subjected to a reaction with mono-6-(*p*-tolylsulfonyl)-β-cyclodextrin (**3**) [2]. To this end, **3** (500 mg, 0.39 mmol) together with potassium iodide (3 mg, 0.02 mmol) and a pyridine aldoxime (474 mg, 3.9 mmol, 10 equiv) or a pyridine ketoxime (369 mg, 2.7 mmol) were dissolved in dry DMF (5 ml). After being stirred at 80 °C under a nitrogen atmosphere, for 12 d in the case of aldoximes and 10 d in the case of ketoximes, the reaction mixture was poured into acetone. The precipitate was filtered off and washed with acetone. Separation and purification of the product was achieved by preparative HPLC.

6-(3-(Oxidoiminomethyl)pyridinium-1-yl)-6-deoxy-β-cyclodextrin (**1b**). Yield: 115 mg (24%); ^{1}H NMR (D_2O, 400 MHz) δ 2.61–2.63 (m, 1H), 2.90–2.93 (m, 1H), 3.41–3.72 (m, 17H), 3.76–4.13 (m, 22H), 4.28–4.34 (m, 1H), 4.96–5.25 (m, 7H), 8.02–8.06 (m, 1H), 8.22 (s, 1H), 8.69–8.73 (m, 2H), 8.99 (s, 1H); ^{13}C NMR (D_2O, 100.6 MHz) δ 59.1, 60.3–62.3, 70.5–73.0, 80.8–83.1, 101.4–101.9, 128.1, 136.1, 140.6, 141.5, 141.7, 143.5; MALDI-TOF MS, *m*/*z* (%) 1223.9 (100%) $[M - O + H]^+$, 1239.9 (29%) $[M + H]^+$; elemental analysis calcd (%) for $C_{48}H_{74}N_2O_{35}\cdot 4H_2O$: C, 43.97; H, 6.30; N, 2.14; found: C, 44.05; H, 6.54; N, 2.04.

6-(4-(Oxidoiminomethyl)pyridinium-1-yl)-6-deoxy-β-cyclodextrine (**1c**). Yield: 275 mg (29%); ^{1}H NMR (D_2O, 400 MHz) δ 2.74–2.78 (d, 1H, J(H,H) = 12.2 Hz), 2.97–3.00 (d, 1H, J(H,H) = 12.4 Hz), 3.40–3.65 (m, 14H), 3.66–4.01 (m, 23H), 4.10 (t, 1H, J(H,H) = 9.8 Hz), 4.62 (t, 1H, J(H,H) = 10.4 Hz), 4.98–5.18 (m, 8H), 8.05 (d, 2H, J(H,H) = 5.9 Hz), 8.23 (s, 1H), 8.61 (d, 2H, J(H,H) = 6.0 Hz); ^{13}C NMR (DMSO-d_6, 100.6 MHz) δ 58.3–60.8, 70.1–

73.5, 80.1–83.7, 100.7–102.4, 116.1, 142.1, 144.7, 153.3; MALDI-TOF MS, *m/z* (%) 1210.8 (13%) $[M - NO + H]^+$, 1223.8 (100%) $[M - O + H]^+$, 1239.7 (88%) $[M + H]^+$; elemental analysis calcd (%) for $C_{48}H_{74}N_2O_{35}\cdot 3H_2O\cdot C_3H_6O$: C, 45.33; H, 6.42; N, 2.07; found: C, 45.48; H, 6.38; N, 2.15.

6-(3-(1-(Oxidoimino)ethyl)pyridinium-1-yl)-6-deoxy-β-cyclodextrin (**1d**). Yield: 123 mg (25%); ^1H NMR (D_2O , 400 MHz) δ 2.21 (s, 3H), 2.59–2.62 (m, 1H), 2.88 (d, 1H, *J*(H,H) = 11.9 Hz, 3.41–3.72 (m, 16H), 3.76–4.04 (m, 20H), 4.08–4.12 (m, 1H), 4.32–4.37 (m, 1H), 4.95–5.26 (m, 9H), 8.06–8.09 (m, 1H), 8.75–8.77 (m, 2H), 9.04 (s, 1H); ^{13}C NMR (D_2O, 100.6 MHz) δ 11.1, 59.2–62.3, 70.7–73.1, 80.7–83.0, 101.3–102.0, 127.6, 139.1, 141.6, 142.2, 143.6, 150.8; MALDI-TOF MS, *m/z* (%) 1237.8 (100%) $[M - O + H]^+$), 1253.8 (19%) $[M + H]^+$; elemental analysis calcd (%) for $C_{49}H_{76}N_2O_{35}\cdot 6H_2O\cdot 0.5C_3H_6O$: C, 43.63; H, 6.60; N, 2.01; found: C, 43.66; H, 6.58; N, 1.88.

6-(4-(1-(Oxidoimino)ethyl)pyridinium-1-yl)-6-deoxy-β-cyclodextrin (**1e**). Yield: 187 mg (38%); ^1H NMR (D_2O , 600 MHz) δ 2.18 (s, 3H), 2.70–2.72 (m, 1H), 2.88 (d, 1H, *J*(H,H) = 11.5 Hz), 3.41–3.66 (m, 14H), 3.68–4.10 (m, 22H), 4.21 (t, 1H, *J*(H,H) = 9.9 Hz), 4.61–4.65 (m, 1H), 4.95–5.16 (m, 9H), 8.17 (d, 2H, *J*(H,H) = 7.0 Hz), 8.62 (d, 2H, *J*(H,H) = 7.0 Hz); ^{13}C NMR (D_2O, 151 MHz) δ 10.1, 59.3–60.8, 70.6–73.1, 81.0–83.1, 101.4–102.0, 122.0, 144.0, 153.0, 155.1; MALDI-TOF MS, *m/z* (%) 1237.9 (100%) $[M - O + H]^+$, 1253.9 (38%) $[M + H]^+$; elemental analysis calcd (%) for $C_{49}H_{76}N_2O_{35}\cdot 5H_2O\cdot 0.5C_3H_6O\cdot 0.5C_2H_5OH$: C, 44.33; H, 6.65; N, 2.01; found: C, 44.15; H, 6.41; N, 1.57.

3-((Hydroxyimino)methyl)-1-(prop-2-ynyl)pyridinium bromide (**6**). A solution of picolinaldehyde oxime [3] (366 mg, 3 mmol) in DMF (1.5 ml) was added over the course of 2 h to a solution of propargyl bromide in toluene (80%, 1.34 g, 1.00 ml, 9.00 mmol) containing solid potassium iodide (50 mg, 0.30 mmol). A precipitate was formed which was filtered off, washed with DMF and acetone, and dried. Yield: 281 mg (39%); mp 167–169 °C; ^1H NMR (D_2O, 400 MHz) δ 3.31 (s, 1H), 5.57 (s, 2H), 8.14 (t, 1H, *J*(H,H) = 6.6 Hz), 8.38 (s, 1H), 8.77 (d, 1H, *J*(H,H) = 8.0 Hz), 9.01 (d, 1H, *J*(H,H) = 5.7 Hz), 9.24 (s, 1H); ^{13}C NMR (DMSO-d_6, 151 MHz) δ 50.9, 73.4, 80.6, 128.4, 133.7, 142.1, 143.3, 144.0, 144.7; IR (cm^{-1}) 2131 (ν (C≡C)); ESI MS, *m/z* (%) 160.9 (100%) $[M - Br]^-$; elemental analysis calcd (%) for $C_9H_9N_2OBr$: C, 44.84; H, 3.76; N, 11.62; found: C, 44.78; H, 4.00; N, 11.68.

General procedure for the preparation of 6-((trimethylsilyl)ethynyl)-formylpyridines. The corresponding 6-bromo-formylpyridine (500 mg, 2.69 mmol) was dissolved in freshly distilled triethylamine (10 ml) under an atmosphere of nitrogen. Copper(I)iodide (51.2 mg, 269 μmol), bis(triphenylphosphine)palladium(II)dichloride (75.5 mg, 108 μmol), bis[(2-

diphenylphosphino)phenyl] ether (57.9 mg, 108 μmol) and ethynyltrimethylsilane (317 mg, 3.23 mmol) were added successively and the resulting mixture was stirred at 25 °C for 18 h. Afterward, the solvent was removed and the residue was purified chromatographically by using hexane/ethyl acetate, 4:1 (v/v) as eluent.

6-((Trimethylsilyl)ethynyl)-2-formylpyridine. Yield 384 mg (71%); R_f 0.62 (hexane/ethyl acetate, 4:1 (v/v)); mp 63–64 °C; ^{1}H NMR ($CDCl_3$, 400 MHz) δ 0.29 (s, 9H), 7.66 (dd, 1H), 7.83 (t, 1H), 7.89 (dd, 1H), 10.06 (s, 1H); ^{13}C NMR ($CDCl_3$, 100.6 MHz) δ −0.2, 97.0, 102.6, 120.7, 131.5, 137.4, 143.8, 153.0, 193.1; IR (cm^{-1}) 2155 (ν (C≡C)), 1726 (ν (C=O)); ESI-TOF MS, *m*/*z* (%) 203.9 (63%) $[M + H]^+$, 225.9 (100%) $[M + Na]^+$; elemental analysis calcd (%) for $C_{11}H_{13}NOSi$: C, 64.98; H, 6.44; N, 6.89; found: C, 65.09; H, 6.48; N, 6.92.

6-((Trimethylsilyl)ethynyl)-3-formylpyridine. Yield 427 mg (78%); R_f 0.53 (hexane/ethyl acetate, 4:1 (v/v)); mp 85–87 °C, ^{1}H NMR ($CDCl_3$, 400 MHz) δ 0.29 (s, 9H), 7.59 (d, 1H), 8.12 (dd, 1H), 9.01 (s, 1H), 10.09 (s, 1H); ^{13}C NMR ($CDCl_3$, 151 MHz) δ −0.32, 100.1, 103.0, 127.6, 130.1, 136.0, 147.9, 152.3, 190.0; IR (cm^{-1}) 2164 (ν (C≡C)), 1686 (ν (C=O)); ESI-TOF MS, *m*/*z* (%) 203.9 (10%) $[M + H]^+$, 225.9 (100%) $[M + Na]^+$; elemental analysis calcd (%) for $C_{11}H_{13}NOSi$: C, 64.98; H, 6.44; N, 6.89; found: C, 65.20; H, 6.59; N, 6.91.

6-((Trimethylsilyl)ethynyl)-4-formylpyridine. Yield 472 mg (81%); R_f 0.45 (hexane/ethyl acetate, 4:1 (v/v)); ^{1}H NMR ($CDCl_3$, 400 MHz) δ 0.29 (s, 9H), 7.59 (d, 1H), 8.12 (dd, 1H), 9.01 (s, 1H), 10.09 (s, 1H); ^{13}C NMR ($CDCl_3$, 151 MHz) δ −0.3, 97.3, 102.7, 121.1, 126.1, 141.8, 144.8, 151.4, 190.8; IR (cm^{-1}) 2161 (ν (C≡C)), 1710 (ν (C=O)); ESI-TOF MS, *m*/*z* (%) 203.9 (100%) $[M + H]^+$; elemental analysis calcd (%) for $C_{11}H_{13}NOSi$: C, 64.98; H, 6.44; N, 6.89; found: C, 64.83; H, 6.46; N, 6.80.

General procedure for the preparation of 6-ethynyl-formylpyridine oximes. The corresponding 6-((trimethylsilyl)ethynyl)-formylpyridine (300 mg, 1.48 mmol) was suspended in water (9 ml) and an aqueous solution of hydroxylamine (50%) (272 μl, 4.44 mmol) was added. The precipitate formed was filtered off after 30 min stirring at rt, washed with water, and dried. Further purification was achieved as described for the individual products below.

6-Ethynyl-2-formylpyridine oxime (**7a**). The crude product was dissolved in THF (10 ml) and tetrabutylammonium fluoride (387 mg, 1.48 mmol) was added at 0 °C. After being stirred for 1 h the reaction mixture was diluted with ethyl acetate (5 ml) and washed with water. The aqueous layer was extracted five times with ethyl acetate. The combined organic layers were dried over sodium sulfate and the solvent was removed in vacuo. Further purification was achieved by recrystallization from a small amount of water. Yield: 121 mg (56%); R_f 0.55 (hexane/ethyl acetate, 1:1 (v/v)); mp 169–171 °C; ^{1}H NMR (DMSO-d_6, 400 MHz) δ 4.38 (s,

1H), 7.55 (d, 1H, J(H,H) = 7.3 Hz), 7.79 (d, 1H, J(H,H) = 7.6 Hz), 7.80 (t, 1H, J(H,H) = 7.7 Hz), 8.05 (s, 1H), 11.81 (s, 1H); ^{13}C NMR (DMSO-d_6, 100.6 MHz) δ 80.6, 82.7, 119.8, 127.4, 137.5, 141.5, 148.4, 152.7; IR (cm^{-1}) 2109 (ν (C≡C)); ESI-TOF MS, m/z (%) 147.0 (6%) $[M + H]^+$, 168.9 (100%) $[M + Na]^+$; elemental analysis calcd (%) for $C_8H_6N_2O$: C, 65.75; H, 4.14; N, 19.17; found: C, 65.53; H, 4.45; N, 18.84.

6-Ethynyl-3-formylpyridine oxime (**7b**). The crude product was purified chromatographically by using hexane/MTBE, 1:1 (v/v) as eluent. Yield: 179 mg (83%); R_f 0.30 (hexane/MTBE, 1:1 (v/v)); mp 190–191 °C; ^{1}H NMR (DMSO-d_6, 400 MHz) δ 4.43 (s, 1H), 7.57 (d, 1H, J(H,H) = 8.1 Hz), 7.98 (dd, 1H, J(H,H) = 8.1 Hz, J(H,H) = 2.0 Hz), 8.20 (s, 1H), 8.73 (s, 1H), 11.68 (s, 1H); ^{13}C NMR (DMSO-d_6, 151 MHz, 100 °C) δ 79.8, 82.5, 126.5, 128.3, 132.9, 141.5, 144.7, 147.5; IR (cm^{-1}) 2099 (ν (C≡C)); ESI-TOF MS, m/z (%) 147.0 (55%) $[M + H]^+$, 168.9 (100%) $[M + Na]^+$; elemental analysis calcd (%) for $C_8H_6N_2O$: C, 65.75; H, 4.14; N, 19.17; found: C, 65.54; H, 4.45; N, 18.76.

6-Ethynyl-4-formylpyridine oxime (**7c**). This compound was pure without further purification. Yield: 198 mg (92%); R_f 0.42 (hexane/MTBE, 1:1 (v/v)); mp 198–200 °C; ^{1}H NMR (DMSO-d_6, 400 MHz) δ 4.38 (s, 1H), 7.58 (d, 1H, J(H,H) = 5.0 Hz), 7.68 (s, 1H), 8.17 (s, 1H), 8.57 (d, 1H, J(H,H) = 5.1 Hz), 11.94 (s, 1H); ^{13}C NMR (DMSO-d_6, 100.6 MHz) δ 80.5, 82.8, 120.2, 124.3, 141.1, 142.3, 146.1, 150.7; IR (cm^{-1}) 2109 (ν (C≡C)); ESI-TOF MS, m/z (%) 147.0 (100%) $[M + H]^+$; elemental analysis calcd (%) for $C_8H_6N_2O$: C, 65.75; H, 4.14; N, 19.17; found: C, 65.71; H, 4.41; N, 19.05.

Cyclodextrins 2a–**2d**. Mono-6-azido-6-deoxy-β-cyclodextrin [4] (300 mg, 0.26 mmol) was dissolved in ethanol/water, 1:1 (v/v) (10 ml) under an atmosphere of nitrogen. Copper(II) sulfate pentahydrate (3.25 mg, 13.0 μmol), sodium ascorbate (10.3 mg, 0.05 mmol), TBTA (6.85 mg, 13.0 μmol), and the respective alkyne **7a**, **7b**, or **7c** (0.31 mmol) were added successively and the resulting mixture was stirred at 25 °C in the case of **2a** and 70 °C in the case of **2b**–**2d**, for 1 to 3 d. After conversion was complete, the reaction mixture was poured into acetone. The precipitate was filtered off and washed with acetone. Separation and purification of the product was achieved by preparative HPLC.

6-(4-(1-(3-((Oxidoimino)methyl)pyridinium-1-yl)-methyl)-1*H*-1,2,3-triazol-1-yl)-6-deoxy-β-cyclodextrin (**2a**). Yield: 193 mg (56%); ^{1}H NMR (D_2O, 600 MHz) δ 2.60 (d, 1H, J(H,H) = 11.7 Hz), 2.71 (d, 1H, J(H,H) = 12.2 Hz), 3.44–3.69 (m, 14H), 3.76–3.99 (m, 24H), 4.22–4.25 (m, 1H), 4.62–4.66 (m, 1H), 4.94–5.16 (m, 7H), 5.97 (s, 2H), 8.05 (t, 1H, J(H,H) = 6.9 Hz), 8.23 (s, 1H), 8.33 (s, 1H), 8.65 (d, 1H, J(H,H) = 8.2 Hz), 8.87 (d, 1H, J(H,H) = 5.6 Hz), 9.11 (s, 1H); ^{13}C NMR (D_2O, 151 MHz) δ 51.5, 55.3, 59.1–60.3, 70.3–73.0, 80.7–

83.1, 101.5–102.0, 127.7, 128.4, 136.1, 139.5, 141.0, 141.7, 142.7, 143.8; MALDI-TOF MS, *m*/*z* (%) 1304.6 (100%) $[M - O + H]^+$, 1320.7 (35%) $[M + H]^+$; elemental analysis calcd (%) for $C_{51}H_{77}N_5O_{35}\cdot H_2O\cdot C_3H_6O$: C, 46.45; H, 6.14; N, 5.02; found: C, 46.54; H, 6.34; N, 5.08.

6-(4-(6-(Hydroxyimino)methyl-pyridine-2-yl)-1*H*-1,2,3-triazol-1-yl)-6-deoxy-β-cyclodextrin (**2b**). Yield 110 mg (33%); ^{1}H NMR (DMSO-d_6, 400 MHz) δ 3.06 (s, 2H), 3.23–3.41 (m, 14H), 3.54–3.65 (m, 22H), 4.08 (t, 1H, *J*(H,H) = 7.4 Hz). 4.29 (t, 1H, *J*(H,H) = 5.3 Hz), 4.49–4.53 (m, 6H), 4.73–4.94 (m, 8H), 5.11 (s, 1H), 5.69–5.75 (m, 14H), 7.74 (d, 1H, *J*(H,H) = 7.7 Hz), 7.92 (t, 1H, *J*(H,H) = 7.8 Hz), 7.98 (d, 1H, *J*(H,H) = 7.7 Hz), 8.11 (s, 1H), 8.51 (s, 1H), 11.76 (s, 1H); ^{13}C NMR (DMSO-d_6, 101 MHz) δ 50.4, 58.9–59.9, 69.7–73.2, 81.0–82.1, 101.4–102.2, 118.9, 119.7, 125.0, 137.9, 146.4, 148.7, 149.9, 152.1; MALDI-TOF MS, *m*/*z* (%) 1291.1 (41%) $[M - O + H]^+$, 1307.1 (19%) $[M + H]^+$, 1313.1 (74%) $[M - O + Na]^+$, 1329.1 (100%) $[M + Na]^+$; elemental analysis calcd (%) for $C_{50}H_{75}N_5O_{35}\cdot 6H_2O$: C, 42.46; H, 6.20; N, 4.95; found: C, 42.47; H, 6.26; N, 4.67.

6-(4-(5-(Hydroxyimino)methyl-pyridine-2-yl)-1*H*-1,2,3-triazol-1-yl)-6-deoxy-β-cyclodextrin (**2c**). Yield 79.4 mg (24%); ^{1}H NMR (DMSO-d_6, 400 MHz) δ 2.96 (s, 2H), 3.31–3.52 (m, 14H), 3.67–3.81 (m, 22H), 4.11 (t, 1H, *J*(H,H) = 8.4 Hz), 4.26 (t, 1H, *J*(H,H) = 5.1 Hz), 4.46–4.57 (m, 6H), 4.69–4.85 (m, 8H), 5.07 (s, 1H), 5.67–5.93 (m, 14H), 8.04 (d, 1H, *J*(H,H) = 8.2 Hz), 8.11 (d, 1H, *J*(H,H) = 8.1 Hz), 8.22 (s, 1H), 8.59 (s, 1H), 8.74 (s, 1H), 11.55 (s, 1H); ^{13}C NMR (DMSO-d_6, 100.6 MHz) δ 50.6, 58.7–60.4, 69.8–73.3, 80.7–83.5, 101.2–102.3, 119.5, 124.5, 128.0, 133.9, 145.6, 146.7, 148.1, 150.5; MALDI-TOF MS, *m*/*z* (%) 1291.2 (49%) $[M - O + H]^+$, 1307.2 (25%) $[M + H]^+$, 1313.1 (85%) $[M - O + Na]^+$, 1329.1 (100%) $[M + Na]^+$; elemental analysis calcd (%) for $C_{50}H_{75}N_5O_{35}\cdot 4H_2O$: C, 43.57; H, 6.07; N, 5.08; found: C, 43.38; H, 6.35; N, 4.90.

6-(4-(4-(Hydroxyimino)methyl-pyridine-2-yl)-1*H*-1,2,3-triazol-1-yl)-6-deoxy-β-cyclodextrin (**2d**). Yield 106 mg (31%); ^{1}H NMR (DMSO-d_6, 400 MHz) δ 2.97 (s, 2H), 3.21–3.47 (m, 14H), 3.58–3.83 (m, 22H), 4.11 (t, 1H, *J*(H,H) = 8.1 Hz), 4.25 (t, 1H, *J*(H,H) = 4.3 Hz), 4.40–4.56 (m, 6H), 4.66–4.95 (m, 8H), 5.06 (s, 1H), 5.67–5.89 (m, 14H), 7.50 (d, 1H, *J*(H,H) = 4.6 Hz), 8.20 (s, 1H), 8.27 (s, 1H), 8.58 (s, 2H), 11.86 (s, 1H); ^{13}C NMR (DMSO-d_6, 100.6 MHz) δ 50.6, 58.6–60.4, 69.8–73.3, 80.8–83.5, 101.3–102.3, 116.2, 119.5, 124.4, 141.4, 146.8, 146.9, 150.1, 150.7; MALDI-TOF MS, *m*/*z* (%) 1291.1 (49%) $[M - O + H]^+$, 1307.1 (100%) $[M + H]^+$, 1313.1 (42%) $[M - O + Na]^+$, 1329.1 (96%) $[M + Na]^+$; elemental analysis calcd (%) for $C_{50}H_{75}N_5O_{35}\cdot 7H_2O$: C, 41.93; H, 6.26; N, 4.89; found: C, 41.84; H, 6.29; N, 4.68.

References

1. Tanner, D.; Wennerström, O. *Acta Chem. Scand., Ser. B* **1983,** *37,* 693–698. doi:10.3891/acta.chem.scand.37b-0693

2. McNaughton, M.; Engman, L.; Birmingham, A.; Powis, G.; Cotgreave, I. A. *J. Med. Chem.* **2004,** *47,* 233–239. doi:10.1021/jm030916r

3. Ginsburg, S.; Wilson, I. B. *J. Am. Chem. Soc.* **1957,** *79,* 481–485. doi:10.1021/ja01559a067

4. Hamasaki, K.; Ikeda, H.; Nakamura, A.; Ueno, A.; Toda, F.; Suzuki, I.; Osa, T. *J. Am. Chem. Soc.* **1993,** *115,* 5035–5040. doi:10.1021/ja00065a012
